# Patterns of mandibular invasion in oral squamous cell carcinoma of the mandibular region

**DOI:** 10.1186/1477-7819-5-12

**Published:** 2007-01-30

**Authors:** Manoj Pandey, Latha P Rao, Shaima R Das, Anitha Mathews, Elizabeth M Chacko, BR Naik

**Affiliations:** 1 Department of Surgical Oncology, Regional Cancer Centre, Thiruvananthapuram Kerala, India; 2 Department of Surgical Oncology, Institute of Medical Sciences, Banaras Hindu University, Varanasi 221005, India; 3 Department of oral maxillofacial surgery, Government Dental College, Thiruvananthapuram Kerala, India; 4 Department of Pathology, Government Medical College, Thiruvananthapuram Kerala, India; 5 Department of Pathology, Regional Cancer Centre, Thiruvananthapuram, India; 6 Department of Oral and maxillofacial Surgery, Amritha Institute of Medical Sciences, Kochi, India

## Abstract

**Background:**

Mandibular resections are routinely carried out for achieving a R0 resection for oral cancers. However, the need of mandibular resection to achieve this has always been questioned. The present study was carried out to define the pattern of mandibular involvement in carcinoma of the mandibular region.

**Patients and methods:**

A total of 25 consecutive patients who had undergone mandibular resection and were found to have mandibular invasion were studied in a prospective open fashion. After decalcification the specimens were serially sectioned at 1 cm interval to identify invasion of mandibular bone. Type of invasion, route of spread and host cell reactions were also recorded.

**Results:**

The mandibular involvement was infiltrative in 14(56%) and erosive in 11(44%). It was cortical in 5(20%), marrow involvement was seen in 15(60%) while 5(20%) had spread through the inferior alveolar canal. Of the 25, 24(96%) lesions were located with in 1 cm of the mandible.

**Conclusion:**

The possibility of mandibular involvement is higher in patients where tumours are located with in 1 cm of the mandible. Involvement of mandible through the canal of inferior alveolar nerve in the present study was relatively high (20%). Therefore it is recommended that before a decision is taken to preserve the mandible it should be thoroughly screened for possible involvement.

## Background

Oral carcinoma of the mandibular region has been defined as carcinoma of the mandibular alveolar ridge, lower buccal sulcus, sublingual sulcus and mandibular retro molar trigone [1]. Lesions occurring in this area often involve mandible mostly by direct extension and seldom by other routes [2,3]. The prevalence of mandibular bone involvement ranges from 12 to 56% [4-7]. The treatment results of these lesions are usually poor with nearly 70% recurring at primary site that ultimately cause death [8,9].

Mandibular involvement in these cases occurs mainly due to direct infiltration of the mandible by tumour. Main route of entry into mandible is reported to be through alveolar crest and lingual cortex if the tumour is located medial to mandible [10,11]. Other routes of infiltration are also described, of which spread though the canal of inferior alveolar nerve is most important in the present scenario where a lot of emphasis is being placed on conserving mandible. Too many mandibles are sacrificed without histological evidence of mandibular invasion [12]. For reasons of quality of life, it is important to preserve the continuity of the mandible, if oncologically safe [13]. The problem with preservation of the continuity of the mandible, is that surgeons often feel uncomfortable because accurate preoperative examination methods are lacking [14].

This study was carried out to determine the route of entry and pattern of spread in to the mandible from oral squamous cell carcinoma.

## Materials and methods

Between September 2000 and May 2001, a prospective open labelled study was carried out to assess the mandibular invasion in patients with oral squamous cell carcinoma located with in 2 cm of the mandible. All patients underwent an orthopantomogram (OPG), and computed tomography of the mandible to detect the bone invasion. A total of 25 out of 51 patients suspected to have clinical or radiological suspicion of mandibular invasion and undergoing hemi or segmental mandibulectomy were included in the present study.

Data on clinical findings, radiological findings and operative findings was collected prospectively on a preset proforma. Grossing of the resected specimens was carried out the day after the surgery. Bits were taken from the tumour and all the inked margins. The specimens were then kept for decalcification and were serially cut at 1 cm interval to determine mandibular invasion. Bits were taken and embedded in paraffin, from which serial 5 μ sections were cut and were stained with haematoxylin and eosin. Beside the pathological invasions of mandible the type of invasion, route of invasion and host cell reaction were noted along with routine tumour characteristics. Involvement of the cortex without involvement of bone marrow was categorised as cortical involvement, while patients with presence of bone marrow involvement were labelled as marrow involvement. Data was tabulated and cross tabulations were prepared.

## Results

Of the 25 patients there were 17(68%) males and 8(32%) females with a mean age of 56.9 years (SD 10.5). Of these, only 7 were habituated to either chewing tobacco or smoking, while one used alcohol. Tumours were located on lower gingiva in 17(68%), buccal mucosa in 7(28%) and on tongue extending to sublingual sulcus in 1(4%) patient, 24(96%) of these were with in 1 cm from the mandible. Other tumour characteristics are detailed in table 1. There were 5(20%) cases of clinical T_1 _lesions, 8(32%) of T_2 _lesions and 12(48%) of T_4 _lesions. Postoperatively all the cases were reclassified as T_4_. Six (24%) had undergone previous radiotherapy and were operated for salvage of residual tumour while rest (76%) had undergone primary surgery.

**Table 1:** Patient and disease characteristics

Characteristic/variable	Grouping	Number	Percentage
Sex	Male	17	68
	Female	8	32
Religion	Hindu	16	64
	Christian	4	16
	Muslim	5	20
Education	Illiterate	2	8
	Primary	14	56
	Secondary	8	32
	College	1	4
Habits	No	7	28
	Yes (Current)	5	20
	Past habits	13	52
Site	Lower alveolus	17	68
	Buccal mucosa	7	28
	tongue extending to sublingual sulcus	1	4
Side	Left	16	64
	Right	9	36
Presenting complain	Ulcer	13	52
	Swelling	8	32
Skin involvement	Positive	6	24
	Negative	19	76
Presence of teeth	Edentulous	11	44
	Dentulous	14	56
Status of tumour	Primary	17	68
	Recurrent	5	20
	Residual	3	12
Nodes	Present	19	76
	Absent	6	24
Clinical tumour stage	T4	25	100
Radiological bone involvement	Present	23	92
	Absent	2	8
Type of mandibular surgery	Segmental resection	4	16
	Hemimandibulectomy	21	84
Previous radiotherapy	Yes	6	24
	No	19	76

**Figure 1:**
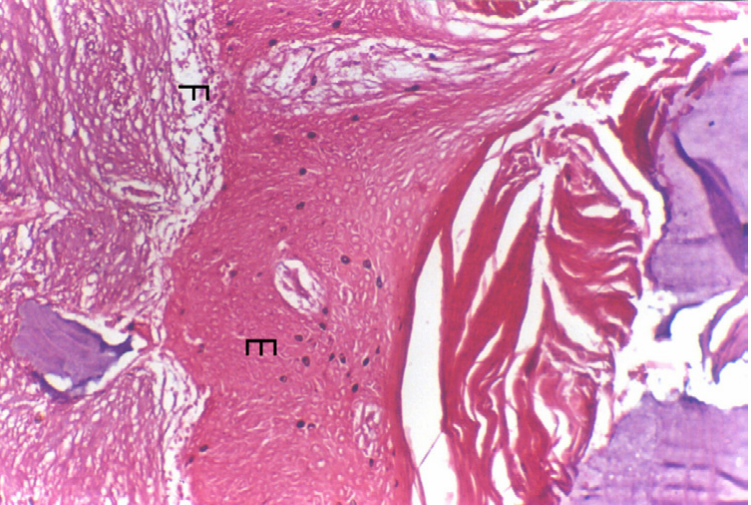
Tumour invasion in mandible in a broad front (E) characteristic of erosive pattern with no bony remnants within the tumour mass tumour cells are separated from the normal bone by a well demarcated fibrous zone (F).

**Figure 2:**
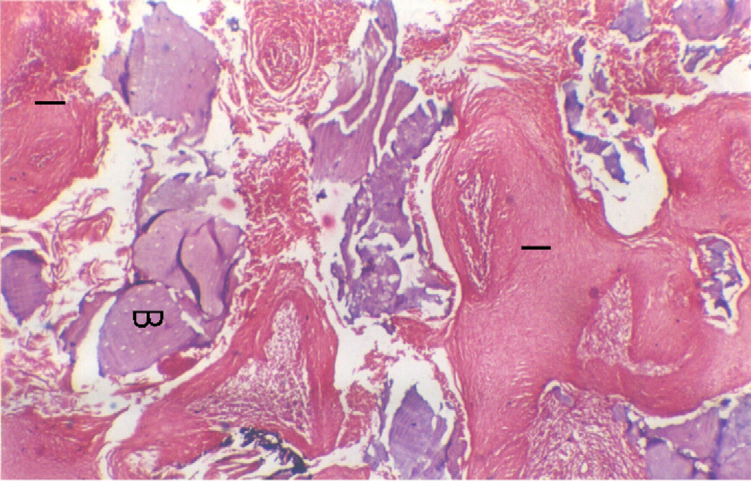
Tumour invasion in the mandible as irregular cords and islands (I) characteristic of infiltrative pattern, partially lysed bone spicules (B) are present within the tumour mass with no clear cut demarcating tissue between tumour and bone.

**Figure 3:**
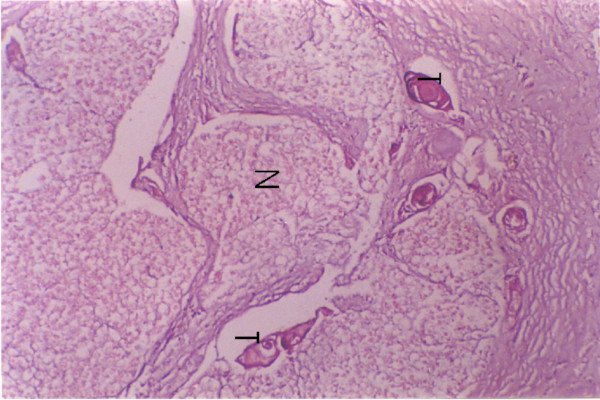
Tumour invasion (T) into the perineural space of the inferior alveolar nerve bundles.

**Figure 4:**
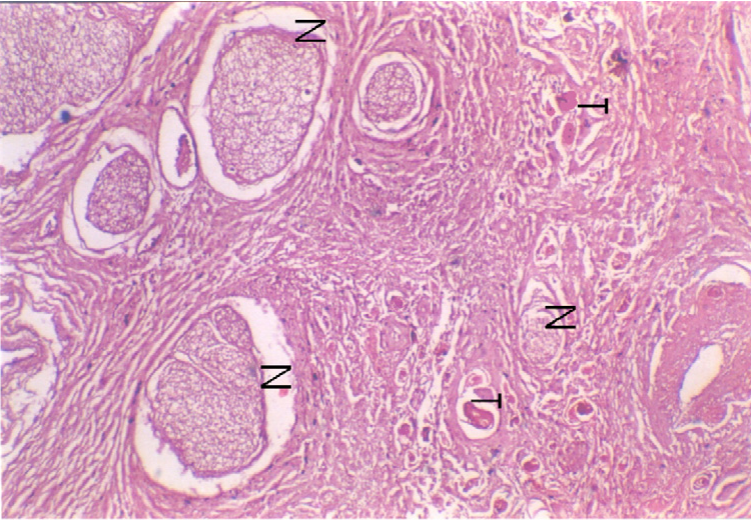
Tumour invasion (T) into the mandible involving the superior portion of the inferior alveolar canal with no invasion of nerve bundles (N).

The mean duration of symptoms was 3.9 ± 2.5 months while the mean depth of invasion was 2.08 ± 0.88 cm. The mandibular involvement was infiltrative in 14 (53%) (Figure 1) while it was erosive in 11(44%) (Figure 2). Pattern of mandibular involvement was characterised as cortical in 5(20%), involvement of bone marrow was seen in 15(60%) while 5(20%) had spread through the canal of inferior alveolar nerve with (Figure 3) or without invasion of inferior alveolar nerve (Figure 4). Depth of invasion was shallow in 8(32%), moderate in 7(28%) and deep in 10(40%). Among the patients who were previously irradiated 2 had perineural spread (2/6 33%), two patients showed subperiosteal fibrosis and one showed new bone formation while two patients had fatty marrow. Three patients each showed infiltrative and erosive pattern in this group.

**Figure 5:**
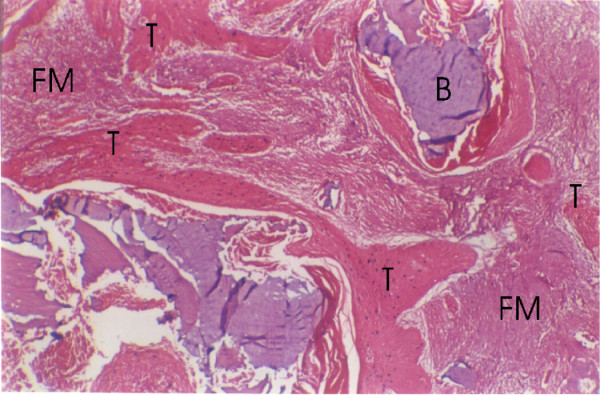
Diffuse irregular tumour infiltrating bone, fibrous marrow (FM) and bony remnants (B).

**Figure 6:**
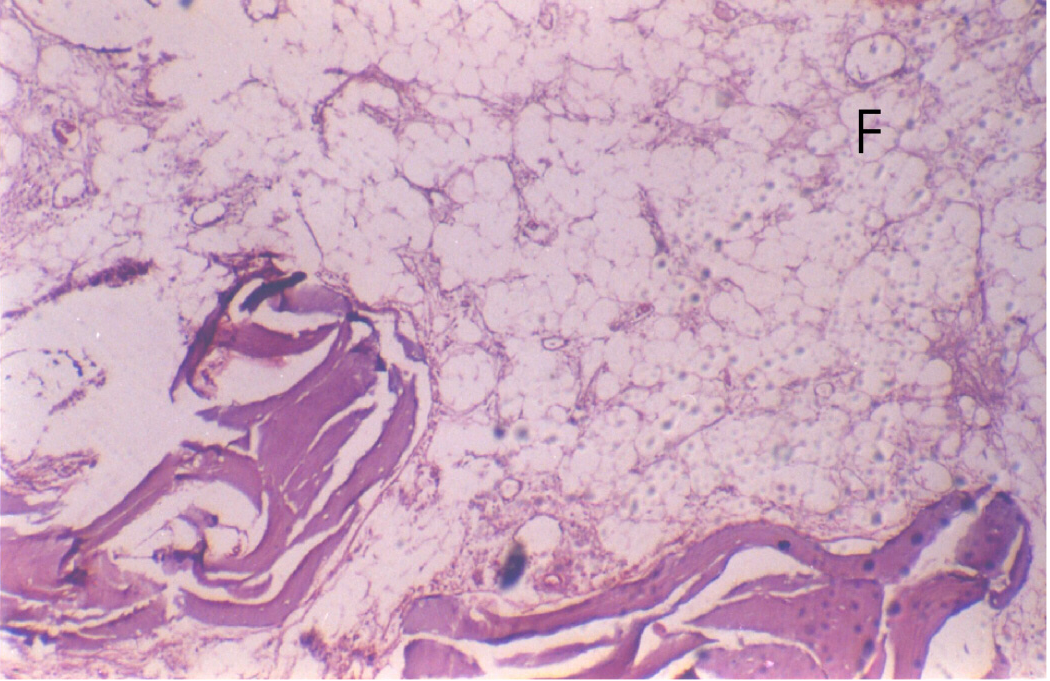
Fatty marrow in post radiotherapy patient.

Fibrosis was commonest host cell reaction identified (Figure 5) followed by inflammation, fatty marrow (Figure 6) and hyperplastic marrow. Infiltrative type of involvement was seen in 10 cases of gingival lesions, 4 cases of buccal lesions while erosive pattern was seen in 7 cases of gingival, 3 cases of buccal mucosa and 1 cases of tongue cancer.

## Discussion

Composite resection of oral cancer with *en bloc *resection of a segment or hemi mandible with neck node dissection was the main stay of treatment for oral squamous cell carcinoma [15]. This had been advocated to allow complete extirpation of tumour which was thought to invade mandible though periosteal lymphatics [16,17]. With increasing understanding of disease spread and biological behaviour of oral cancer it is now clear that majority of the spread to mandible occurs by direct infiltration of the tumour through alveolar ridge or lingual cortical plate [10,18]. It has also been noted that tumours enter at the point of abutment which in both edentulous and dentulous mandible is the junction of reflected and attached mucosa. It has always been believed that mandible could be preserved if there is normal tissue intervening between tumour and the bone. However, in our study we have found a high incidence of spread (20%) through the canal of the inferior alveolar nerve, which often exclude the preservation of mandible. In these cases it is often very difficult to assess the involvement of mandible preoperatively, and subsequent conservation of mandible can lead to positive resection margins if the canal has not been excised. It has also been shown that invasive pattern of disease is evident at shallower depths, [18] however; no such association could be identified in the present study. Dubner and Heller (1993) reported a significantly higher local recurrence rates following marginal resection of mandible which was independent of size of the primary and involvement of the nodes [19]. They further noticed that neither mandibular invasion by tumour nor addition of radiotherapy affected recurrence rates. Similar results have been seen in study of Ord *et al*, [12]. This study being a short outcome study aimed at identifying the invasion pattern follow-up information was not available at the time of reporting however, we are making an effort to collect this information and hope to correlate the mandibular bone involvement, route and type of spread with patterns of failure in future. The present study contains the cases with previous radiotherapy and recurrence and hence the power of the study is weak.

## Conclusion

The results of our study suggest careful preoperative examination of the mandible to rule out bone invasion. We propose that spread through the canal of inferior alveolar nerve or the nerve itself may be the cause of increase rate of local recurrence seen after mandibular conserving surgery.
